# *In plastico*: laboratory material newness affects growth and reproduction of *Daphnia magna* reared in 50-ml polypropylene tubes

**DOI:** 10.1038/srep46442

**Published:** 2017-04-20

**Authors:** Marek Cuhra, Thomas Bøhn, Petr Cuhra

**Affiliations:** 1 Norwegian Institute of Marine Research, Marbank, Forskningsparken, Sykehusv. 21, 9019 Tromsø, Norway; 2 Institute of Farmacology, Faculty of Health Sciences, University of Tromsø, P.O. box 6050 Langnes, 9037 Tromsø, Norway; 3 GenØk – Centre for Biosafety, The Science Park, P.O. box 6418, 9294 Tromsø, Norway; 4 Czech Agriculture and Food Inspection Authority, Za Opravnou 6, 150 00 Praha 5, Czech Republic

## Abstract

Plastic laboratory materials are found to affect vital parameters of the waterflea *Daphnia magna*. The main responsible factor is defined as “newness” of the materials. Juvenile *D. magna* were raised individually in; a) new laboratory-standard 50 ml polypropylene tubes, and; b) identical tubes which had been washed and aerated for several weeks. Newness had significant effects on growth and fecundity of *D. magna*. New tubes caused delayed maturation, reduced reproduction and reduced growth when compared to washed and re-used tubes of the same commercial brand. The findings indicate that newness of tubes has inhibiting or toxic effects on *D. magna*. Often laboratory plastics are intended for single-use due to sterility demands. Newness might be an important confounding factor in research results and should not be disregarded. Disposable plastic utensils may come with a seemingly ignored cost and induce adverse effects in biological test-organisms and systems. The presented findings accentuate continued need for general awareness concerning confounding factors stemming from material laboratory environment. Based on the present findings the authors suggest that plastics intended for use in sensitive research may need to be *washed and aerated* prior to use.

The phenomena of leaching plasticizers^1^, bioaccumulation of undesirable ingredients from plastic-resin^2^ and even presence and mobility of potentially toxic compounds in plastics used in surgical work, transfusions and related medical treatment^3^,^4^ have been well documented in the past. Recent reports on confounding or even disruptive effects of common plastic utensils used in experimental research highlight the need for continued awareness amongst laboratory professionals^5^,^6^,^7^,^8^,^9^.

The term “plastic” encompasses a large variety of products made from synthetic polymer resins such as polyethylene, polypropylene, polyvinylchloride and numerous other compounds, additives and combinations. Such materials are increasingly used in all aspects of society and daily life as well as in research. It has been estimated that the annual global production of various plastic items is approximately 300 million tons^10^, equaling nearly 40 kg for each inhabitant on our planet. An unknown quantity of this annual consumption is used in laboratory research facilities, primarily as disposable single use items. Published research from the previous five decades has shown that chemical leachates from laboratory plastics and medical plastics can have confounding or disruptive effects on outcomes of experiments as well as adding toxic hazards in treatment of patients (summarized in Table 1). An extensive review of 120 peer-reviewed publications^11^ summarized effects of various plastics on health of laboratory animals and humans and highlighted bisphenols and phthalates as two families of chemical plasticizer additives known to contaminate research materials. Throughout our review of available literature we find only sparse information on how various plastics and constituents of plastics affect biological processes, metabolic processes and overall performance of various organisms. We find that surprising, given the fact that such materials are omnipresent in not only laboratory environments but in most other environments as well, notably in the oceans^12^.

In a recent paper the authors R.P. Gale & H.M. Lazarus present a new technical term to describe a dominant material aspect of modern laboratory research; they suggest “*in plastico*” as an expression to supplement the well established terms *in vitro, in vivo* and *in silico*^13^. We find this new term highly relevant, acknowledging that an increasing part of analytical work, purification, storage, incubation as well as uncountable other laboratory procedures are no longer *sensu strictu* performed *in vitro* (the Latin term which means *within the glass*) but instead are taking place within synthetic plastic polymer environments, inside tubes, wellplates, dishes, tips and other such plastic ware. Although polypropylene as a material for laboratory utensils has been considered not to contain additives potentially problematic for laboratory work^11^, this assumption is contradicted by evidence of contaminants leaching into liquid contained in polypropylene centrifuge tubes^14^. This phenomenon was elegantly detected by spectroscopic measurement of qualitative shifts in the liquids held inside the tubes, indicating increased concentration of macromolecules over time.

The present study investigates similar types of polypropylene centrifuge tubes, which are typical disposable commercial plastic products widely used in laboratory research. These standard 50 mm conical polypropylene screwlid centrifuge tube (50 ml-CPPT tube) are commonly known amongst laboratory professionals as “the Falcon-tube”, although manufactured not only by the BD-Biosciences-Falcon Company but also several other commercial producers. We noticed that such 50 ml-CPPT tubes typically have a distinct smell of “new car” when freshly unpacked and opened. This smell subjectively diminishes after several hours when the tubes are left open to aerate.

In 1971 the author K.P. Shea^15^ reflected upon the well-known phenomenon of “the new car smell” and attributed this to volatile plastic compounds such as plasticizers and stabilizers which were released in the confined atmosphere of the car cabin. The author related these additives of common plastics in the automotive interior to the possibly toxic components of PVC blood transfusion bags, at the time suspected of provoking a sometimes fatal medical complication known as “shock lung” in patients receiving donor blood. This complication was discovered at a time when US military hospitals in Vietnam were processing large quantities of battlefield carnage, which facilitated gathering of data from experience in the field. These findings brought a new understanding of how certain plasticizers, previously thought to be *inert,* can escape into fluids or into the air and cause (severe) medical complications.

Producers of laboratory plastics demonstrate awareness that certain qualities or attributes of their products can influence the outcome of the intended use in an undesirable way. Products are manufactured to specific standards and certified with declarations, which describe and guarantee their quality, physical properties and chemical properties. Notably, a major producer of laboratory plastics offers an online catalogue of a wide range of goods of such plastic utensils, including one of the brands of 50 ml-CPPT tubes tested here. The producer gives advice to researchers through the online advertizing material and explicitly states that; “[…] *you should test the sample, not the tube*”^16^. Furthermore, the producer states that tubes are guaranteed to “[…] *meet bioanalytical-grade requirements*” (and to) “[…] *provide unsurpassed performance in critical research applications*”. The tubes are described as having “*consistent biological and physical properties*” and that the actual plastic material (resin) is “*non-toxic and selected via an intense array of US Pharmacopoeia toxicity tests*”^16^. It is of utmost importance that researchers and other users of laboratory materials can fully trust such marketing reassurances.

Anecdotal evidence from colleagues at our analytical laboratories (at the UiT - The Arctic University of Norway) indicates that cultures of bacteria are affected by choice of holding container, with negative effects on microbial growth attributable to specific brands of plastics. Knowledge of such potentially confounding factors and disturbing effects from specific materials has traditionally been transmitted orally as internal workplace information amongst laboratory professionals, typically as advice and recommendations. Such findings should be investigated further, properly documented and finally communicated according to operating procedures and systematic routines. And importantly, the findings must be shared amongst practitioners of our profession.

We acknowledge indications such as those presented in a 2009 Senior Honors Thesis^17^ by Liberty University student D.L. Ortiz, who found that (1) bioactive compounds leached from heat-treated polycarbonate water-bottles and subsequently (2) influenced embryonic development of zebrafish *D. rerio* and 3) induced high mortality.

In the present work we have reared newborn individuals of *Daphnia magna* (waterflea) in 50 ml-CPPT tubes, measuring several biological performance parameters as indications of effects from the holding environment. *D. magna* is a versatile and well-established biological indicator for toxicological and ecotoxicological testing and is routinely used in assessment of chemicals and materials^18^,^19^,^20^. In previous testing we have used *D. magna* in acute and chronic (life-long) testing of dissolved toxins in water^18^, and for effects of various types of materials given as feed in experiments involving 160–300 individually reared daphnids^21^,^22^,^23^. In those experimental set-ups *D. magna* were reared from new-born age in 100 ml laboratory standard borosilicate glass beakers.

It is assumed that the olfactory indication perceived as “smell of new car” noticed in new 50 ml-CPPT tubes indicate a presence of volatile chemicals in the tubes. The experimental design aims at disclosing whether this newness of the tubes may influence life history traits of *D. magna* reared in such environment.

Tested hypothesis (H_0_): No significant effects on survival, growth, age at maturation or fecundity will be registered in *D. magna* reared within the microenvironment of laboratory-standard 50 ml-CPPT tubes which are either; a) new, or; b) washed and re-used.

## Materials and Methods

The experiment was performed at GenØk - Centre for Biosafety at the laboratories for Farmacological and Medical research (UiT - The Arctic University of Norway) in June 2011. The laboratory has performed regular short-term toxicity studies and long-term chronic exposure studies in the common waterflea *D. magna* in the years and months leading up to the present experiment^18^,^21^,^22^,^23^.

Individual plastic tubes were tested as singular microenvironments for holding and cultivating individual *D. magna*. Each individual 50 ml-CPPT tube represented one experimental unit.

### Endpoints and statistics

Survival, age at maturation, growth and fecundity were registered in established standardized procedures^18^,^20^,^21^,^22^. Registration of survival, maturation was done daily. Maturation was registered as a) ovogenesis, defined as first appearance of eggs in brood-chamber, and b) occurrence (birth) of first live juveniles. Quantification of per-capita reproduction by counting viable juveniles, aborted juveniles and aborted eggs and measurements of body length was entered in Excel software spreadsheets and analyzed in SPSS, Systat and R statistics analysis software. Binary variables *Survival* and *Age at maturation* were analyzed with Cox Proportional Hazards (CoxPH) test, and continuous variables *Growth* and *Fecundity* (number of live young and abortions) were analyzed with one/two-ways ANOVA (the latter with *Brand* and *Wash* as independent variables).

### Holding vessels

Experimental unit categories differ in; i) Brand of holding containers defined as brand-A versus brand-B and; ii) Age of holding containers defined as *new*, freshly out of the box, versus *washed*.

Two common commercial brands of transparent polypropylene 50 ml centrifuge tubes (50 ml-CPPT tubes) were tested: Brand-A = BD-Falcon type 352070, batch no 8214722 (Becton, Dickinson and Company, 1 Becton Drive, Franklin Lakes, New Jersey, 07417 United States). Brand-B = VWR 525-0156(21008–242) batch no 9197-943CC-943D (VWR, Radnor Corporate Center, Building One, Suite 200, 100 Matsonford Road, Radnor, PA 19087, USA). Both brands have similar physical appearance and dimension (114 mm long, 28 mm diameter). Both brands have similar polyethylene (HD-PE) screwlids with differing colour of material; blue (brand-A) and purple (brand-B).

Both brands are packaged in sterile lots of 25 or 50 pieces, sealed in polyethylene bags. The material in BD-Falcon 50 ml-CPPT tubes (brand A) is specified to be nonpyrogenic polypropylene. There is no information on the material quality of the VWR brand of 50 ml-CPPT tubes (brand B).

New 50 ml-CPPT tubes (hereafter: new tubes) were taken a few hours prior to the start of the experiment from the supply stream of the distribution facility for medical and laboratory utensils at the University Hospital MH-building, UiT (n = 25 of each brand). The tubes were left unopened until filled with the holding medium for the experimental animals. All tubes were taken from a supply stream which has a high degree of quality assurance; 1) the providers are all certified companies dealing with high-grade laboratory equipment, 2) the facilities are strictly controlled and accessible only via locked doors. There are highly qualified personnel in charge of re-stocking and systematic overview of supplies, and 3) all materials have been taken out of labeled boxes and labeled bags with producer logo, producer labels and other written information which guarantees that these products are authentic. Furthermore, 4) all materials were handled only by the authors, from the storage facilities all the way through transport and unpacking and use in the lab. Thus we are confident that these products are authentic, as indicated by the batch-specifications.

The aged, used, aerated & washed 50 ml-CPPT tubes (hereafter: washed tubes) were used for an average period of 3 months prior to coming into the experiment, primarily for centrifuging of algal culture in the operating procedure to concentrate and purify feed for daphnia (n = 25 of each brand). These used tubes were washed using a standard procedure using hot water and manual mechanical cleaning followed by rinsing. This cleaning clears such tubes from visual residues of any contamination. No detergent was used in the washing.

### Experimental animals

*D. magna* neonates (juvenile animals = juveniles) less than 24 hrs old were harvested from 70 individually kept mother animals and pooled in a large borosilicate glass petri dish from where they were individually transferred with glass pipette with internal diameter 2 mm and randomly distributed as experimental units by lottery-method. Each animal was kept as an independent experimental unit.

The holding-vessels were filled with 45 ml of holding medium prepared as described below. Juveniles were distributed one in each tube and the tubes were immediately closed airtight. Only female juveniles participated in the experiment.

The experimental animals were of the local “Tromsø-Clone” which has been kept and cultivated in mainly 4-liter borosilicate glass beakers at the UiT facilities since 2007. The clone originates from the *D. magna* laboratory at the Department of Biology, University of Oslo, P.O.Box 1066, Blindern, NO-0316, Norway. The cultivated daphnia were kept in fully synthetic media in the glass beakers and were regularly fed a diet of *Selenastrum sp*. unicellular green algae cultivated in fully synthetic growth medium in artificial lighting. Mature algae cultures were concentrated by dual sequence of centrifuging and subsequent washing in de-ionized tap water. Cultures were quantified by spectrophotometric light loss at 660 nm wavelength^24^, labeled and stored dark at 4 °C. Between experiments feeding of mixed populations of adults and juveniles (10–100 adults in each 4-litre beaker) was done manually based on visual indication (water colour) to compensate for the depletion of the holding medium. During experiments spectrophotometric quantification ensures precise dosage for individually kept animals. For long periods of time the successive generations of the *D. magna* clone are kept at constant 24/7 artificial light, resembling the outdoor solar summer-situation at our latitude.

### Holding medium

The experiment was conducted in the fully synthetic holding-medium Elend-M7^20^. As standard the medium was prepared in the laboratory from stock-solutions kept in glass bottles with plastic (HDPE and PP) screwlids and de-ionized water from the university source. The stock-solutions are prepared from laboratory grade chemicals (typically 99% or higher purity), which are kept for years at room temperature in plastic containers with plastic lids (primarily HDPE and PP plastics). The medium was prepared in 100-liter HDPE plastic vats with submersible stainless-steel magnetic stirrers at the time of the experiment, those plastic vats had been in use for approximately 12 months. ADaM synthetic medium^25^ was used in the years 2006–2010. Since 2010 the M7 medium was predominantly used.

The medium was not renewed during the duration of the experiment. Algae culture solution of *Selenastrum sp*. was added at approximately 0.5 mg C/50 ml to the holding medium, ensuring a sufficient initial amount of feed. Algae concentration was quantified via spectrophotometric measurement and defined as nominal amount of organic carbon. Feed was supplemented through regular feeding with algae feed-solution of known density via precision micropipette.

Individual experimental units were labelled and randomly assigned location within experimental setup consisting of slotted isopor plates. Initial location holding containers on individual plates and subsequent relocation placement and rotation of isopor plates within the experimental area was randomized. Randomization of position for each experimental unit was performed as relocation within experimental set-up following regular endpoint registration.

### Laboratory conditions

The physical parameters dissolved oxygen and pH in holding medium were measured regularly and found to be within the range of acceptance as defined by the OECD-211 Guideline for Testing of Chemicals: *Daphnia magna* reproduction test^20^. Oxygen levels were measured with special attention to tests in capped and airtight tubes. Sacrificial tubes for measurement of oxygen were included for this purpose. Sufficient levels of oxygen saturation were measured, average 8.1 ppm O_2_, (min. 7.4–max. 9.1). Measurements of pH showed an average of 9.7 with some variation (min. 9.3–max. 10.1), the alkalinity being due to the hardness of the fully artificial medium. Uniform constant artificial lighting 24/7 from standard fluorescent tubes was provided, emulating natural lighting in the reproductive season at our geographic locality. Laboratory temperature was monitored regularly (21.9–23.6 °C).

Body size of individual animals was determined from digital imagery of live animals transferred into an adapted micro-environment and photographed through a Leitz fixed magnification loupe fitted with a Nikon D300 high-resolution digital camera for subsequent measuring of carapace length (anterior extreme of head-shield to base of caudal spine) according to standardized procedure using Wayne-Rasband Image-J software^26^ calibrated to an Agar-L4078 scale.

All handling of experimental animals was performed by glass pipettes with smooth rounded edges, internal Ø = 4 mm. Randomly assigned experimental units were manually taken from position at Styrofoam plate in main experimental setup. Experimental animals were individually translocated to a 100-ml borosilicate glass beaker containing the same type of medium with identical temperature and concentration of algal feed as the holding environment of the experimental unit. The mother animals were successively kept in this temporary holding environment in random order while the medium of the experimental unit was filtered through a washed 250 nm plankton-netting. Immediately after filtering the liquid medium and the experimental animal was returned to the experimental unit. The experimental unit was returned to the Styrofoam-plate in a randomized position. Exuviae and reproductive outcome was washed from the netting into a petri dish and inspected under a Leitz 10x magnification stereo loupe. All reproductive outcome was counted and categorized as either; live juveniles, dead juveniles (aborted juveniles) or aborted eggs. Embryonic stages beyond the egg-stage were defined as aborted juveniles and not checked for heart movement. More details on specific and general aspects of the GenØk laboratory routines for *D. magna* Studies are described in previous publications^18^,^20^,^21^,^22^.

## Results

### Survival

The survival was high in all groups (>91%) and no differences between the brands or treatments (washing) were detected.

### Age at maturation

**Figure 1:**
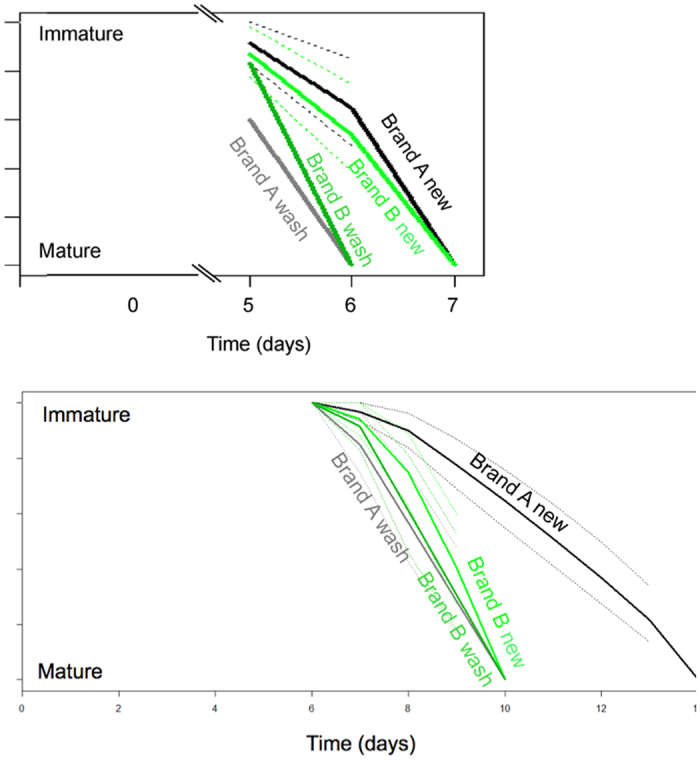
Age at maturation with 95% confidence intervals (when data were sufficient) for *D. magna* reared in new and washed plastic tubes from brand-A and brand-B respectively. Upper panel shows maturation as age at first egg observed in the body cavity of the animal. Lower panel shows age at first juvenile born.

Animals from the new brand-A tubes showed delayed age at maturation, both for the production of visible eggs (ovogenesis) (Fig. 1, upper panel, p < 0.001, CoxPH test) and as live offspring (juveniles) (Fig. 1, lower panel, p < 0.001, CoxPH test) compared to animals living in washed brand-A tubes. For the brand-B tubes, age at maturation for ovogenesis was also delayed in the new compared to washed tubes (Fig. 1, upper panel, p = 0.004, CoxPH test). For age at maturation for juveniles, however, no significant differences between new and washed tubes were observed (Fig. 1, lower panel, p = 0.39, CoxPH test).

An interesting observation was that two animals in new brand-A tubes produced eggs without giving birth of juveniles before seven days later. This indicates that the first eggs were aborted, but that successive reproduction was successful.

### Reproduction

**Figure 2:**
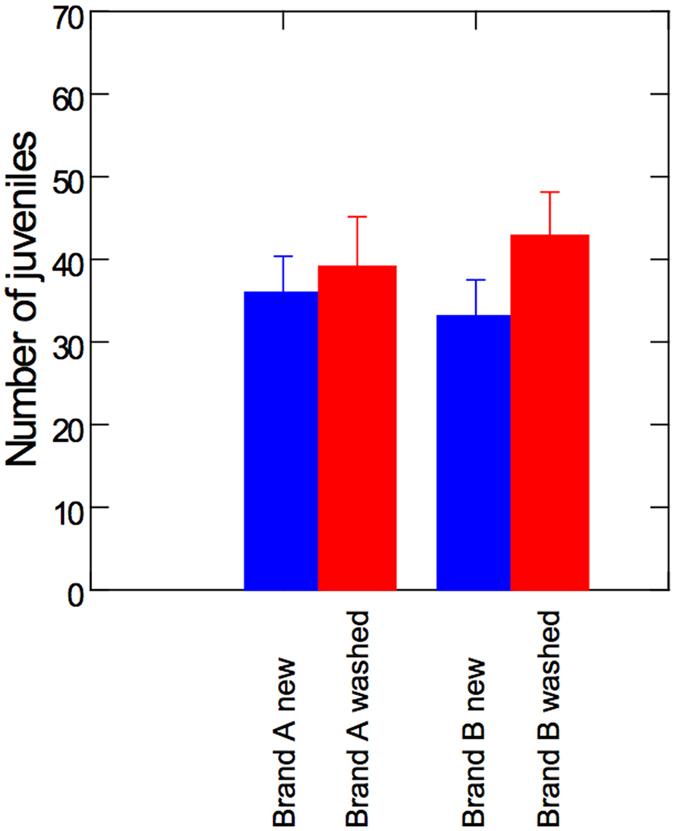
Per capita fecundity with S.E. of *D. magna* reared in new and washed plastic tubes from brand-A and brand-B, respectively.

Animals reared in new brand-A tubes showed no significant difference in reproduction from onset of reproduction to age 15 days (Fig. 2, F = 1.31, p = 0.265, ANOVA), compared to animals reared in washed brand-A tubes. However, animals reared in new brand-B tubes showed significantly reduced reproduction from onset of reproduction to the age of 15 days (Fig. 2, F = 5.33, p = 0.032, ANOVA), when compared to animals reared in washed brand-B tubes. Abortions, i.e. number of dead eggs, embryonal stages or dead juveniles, were somewhat higher in animals that lived in new plastic tubes as compared to animals that lived in washed tubes. However, these differences were not significant (p > 0.225, ANOVA).

Two-ways ANOVA with *Brand* and *Wash* showed that washing was a significant factor to improve fecundity of the animals (F = 5.57, p = 0.02). The *Brand* of the tubes was not a significant factor (p = 0.92) and there was no interaction between *Brand* and *Wash* (p = 0.49).

### Growth

**Figure 3:**
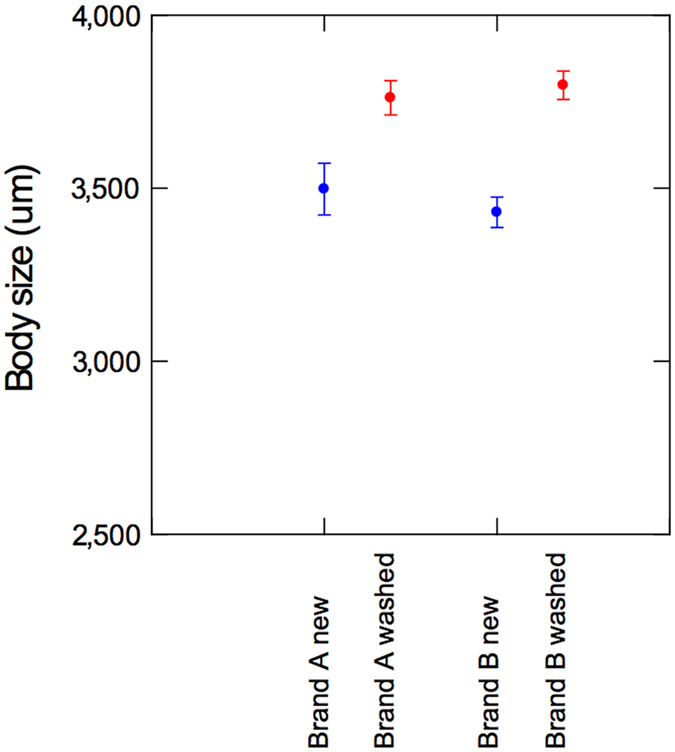
Individual body size with S.E. of *D. magna* reared in new (N) and washed (W) plastic tubes from brand-A and brand-B, respectively.

Animals reared in new brand-A tubes and new brand-B tubes were significantly smaller than animals reared in washed tubes of same brand (Fig. 3, p = 0.008 and p < 0.001, respectively). Two-ways ANOVA with *Brand* and *Wash* showed that washing was a significant factor that reduced body size of animals reared in new plastic tubes (F = 33.74, p < 0.001). The brand of the tubes was not a significant factor (p = 0.58) and there was no interaction between *Brand* and *Wash* (p = 0.35).

## Discussion

Incubating *D. magna* in 50 ml-CPPT tubes demonstrated that onset of reproduction was significantly delayed in new tubes, compared to animals reared in washed tubes of the same brand. Furthermore, both reproductive output and growth was significantly reduced in new tubes, compared to washed tubes of the same brand.

The results indicate significant negative effects attributable to the “newness” of these common laboratory utensils. The unknown causative factors of these significant biological effects in *D. magna* growth, maturation and reproduction may be assumed to be of chemical or physical character and associated with the plastic polymer material of the holding vessel. In this study, we were not able to determine whether this toxic effect is direct or indirect. Indirect effects could stem from changes in the green algae food, or other factors which could reduce the overall environmental quality for the animals. However, we argue that the observed delays of maturation and depression of growth are due to direct toxic effects, as this is supported by previously reported findings:

Importantly, Xu and colleagues have presented results which indicated that phthalic acid leaches from the walls of 50 ml-CPPT tubes^14^. Previously, Mayer and Sanders have demonstrated that phthalic acid esters (DBP, DEHP) have toxic effects in *D. magna* and reduce its reproduction^2^. The work by Xu *et al*. indicated that by leaking from interior tube-wall material of 50 ml-CPPT tubes, increasing concentrations of small molecules alter the spectroscopic qualities of liquid in the tubes. This was quantified with standard spectroscopic methods. The research indicates that the leachates are heterogeneous mixtures of small molecules with the main component probably consisting of phthalic acid and its derivatives. The study by Xu *et al*. did not provide brand-names nor other details on the type of polypropylene centrifuge tube tested. Also, the authors assume that in most cases, leaching of compounds from such plastics represents negligible amounts, which are unlikely to be toxic to humans but still may hamper experimental results^14^. Our results support the second part of that assumption. The authors also conclude that spectroscopy may be used as a simple and fast way to detect leaching contaminants in plastic containers used, both in daily life and laboratory situations.We present *D. magna* testing as a supplementary method to further investigate possible biological effects of water-soluble leachates in plastic holding environments. We also refer to our previous research in which we have used this model in assessment of toxicity of chemicals and toxins at low ppm-levels, in life-long exposure asseys^18^,^27^. *D. magna* is a robust and reliable biological indicator which can be used beyond traditional use in toxicological and ecotoxicological testing. Such testing in *D. magna* can supplement established biological safety testing methods for medical plastics employing human cell lines^3^ or experimental methods using *in vivo* implantation of plastics into rabbit muscle and rat subcutaneous tissue^28^. We see that continuing quality control of plastic utensils is important and we suggest that the method presented here by using the plastic utensils as enclosed holding environments for a known biological indicator organism, could be developed further. New methods should not be restricted to aquatic indicators such as crustaceans or algae, but could also employ terrestrial invertebrates, microorganisms, etc.

Mayer & Sanders showed that phthalates (phthalic acid esters) used as plasticizers are in a family of plastic component chemicals that can form a substantial part of specific plastics constituents (up to 60% in some types of plastics). Phthalates are used as plasticizer in materials such as PVC^2^ and experimental work has shown that up to 34% of PVC plastic material (by weight) consists of phthalates which can leak from the plastic material over time^29^. Phthalates were identified as environmental contaminants following their unexpected discovery in environments such as soils, tissues of cattle and tissue of deep-sea jellyfish. Also, Mayer & Sanders conducted experiments with low concentrations of ^14^C labelled phthalates in water and found these to accumulate in aquatic organisms such as daphnids, insect larvae and fish^2^. Although *acute toxicity* of the tested phthalates was low in *D. magna* (meaning that LC_50_ values = lethal concentrations, were found to be high) the authors found that concentrations 700–3300 times below such high lethal concentrations would negatively impair reproduction in long-term studies. Thus, the authors demonstrated inhibition of reproduction at levels orders of magnitude lower than acute toxicity (LD50)^2^.

The mentioned findings that low concentrations of phthalates significantly impaired *D. magna* reproduction, are relevant in the context of our findings and support the findings of Xu *et al*.^14^. It is thus justified to suggest that the phthalate chemicals found by Xu *et al*. to be leaking into water stored in 50 ml-CPPT tubes could also be present in the 50 ml-CPPT tubes which we have tested.

On the other hand, we have to mention that there could be also other reasons for toxicity. According to the Borealis position^30^, phthalates which are mostly used in production of polypropylene are Bis(2-ethylhexyl) phthalate (DEHP), Dibutyl phthalate (DBP) and Diisobutyl phthalate (DIBP). If completely surviving the polymerization process, the used phthalates could theoretically be present in concentrations of about 1 mg/kg in the final pellets. However, test results have shown phthalate values not exceeding 0,15 mg/kg PP and often even below the threshold of the analytical method of 0,01 mg/kg PP. In addition, phthalates have limited solubility in water – it varies from 0,3 mg/l for DEHP, 1 mg/l for DBP to 13 mg/l for DIBP. It means, that possible concentrations in water have to be very low. The fate of phthalates and their leaching from polypropylene CPPT tubes should be studied. In addition, it is also necessary to take into consideration that determination of phthalates is very difficult due to their presence in almost all labware and lab environment so to obtain correct analytical results is a big challenge.

In spite of the fact that phthalates are a possible casual agent for the observed toxicity of CPPT tubes, the toxic effect could be caused by other component presents in CPPT tubes material and those should also be studied. The further components include all possible compounds which can be in PP material - monomer (propylene) and/or its oligomers and also components from catalyst which consists from different metallics and or organometalics compounds used in polypropylene production, e.g. those based on Titanium, Aluminium, Zirconium and Hafnium elements. Also phthalic acid should be taken into consideration as it is mentioned in Xu *et al*. study^14^. Due to the better water solubility of phthalic acid than of phthalates, it could be leached into the water medium more easily than phthalates. Unfortunately, relevant toxicological data are not available for this substance.

Also the typical “new car” smell of plastics including 50 ml CPPT tubes is probably not caused by phthalates, because of their low volatility connected to their high boiling point (e.g. 340 °C for DBP, 320 °C for DIBP, and 385 °C for DEHP). If phthalates are not the cause of the “new car” smell there must be something else responsible, present in PP – either propylen monomer or oligomers or other components from catalyst. Although presence of phthalates in PP material could explain the observed effects, further studies should be performed to clarify the exact chemical composition and concentration of the leachates. We must also comment on the fact that the commercial manufacturer of one of the brands of plastic tubes that have been tested in this study has presented reassurances concerning the quality of the specific 50 ml-CPPT tubes and the chemical purity of the polypropylene plastic material. As quoted in the introduction of this paper, the manufacturer presents advertising catalogues that explicitly state that the material quality of the 50 ml-CPPT products will not influence biological experiments performed within that environment. This is contrasted by the present results which indicate significant measurable effects from new 50 ml-CPPT tubes in general and the mentioned commercial brand specifically (brand-A, BD-Falcon). Thus, we conclude that the quality reassurances published by that specific commercial manufacturer, should be revised.

The common water-flea *D. magna* is a recognized indicator organism routinely used in ecotoxicological testing and in evaluation of material quality. Results from tests in *D. magna* are likely representative for other cladoceran species and indicative for a wider range of aquatic organisms. We suggest that further research should investigate whether the results reported here have relevance in other biological systems or in human models such as laboratory cell-lines.

In our daphnia laboratory we attempt to uphold homogenous predictable and precisely controlled conditions for the experimental animals by strictly monitoring and regulating such external factors as light, temperature, water-quality and risk of contamination. However, in doing so, we rely on innumerable plastic vessels and structures, which we routinely use in our work and which may present confounding factors, especially when investigating potential effects of other plastics. Such items include tubes, tips and the plastic containers with laboratory grade chemicals, which in turn are used to produce synthetic lake-water and nutrients for the green algae, which are subsequently fed to the daphnia. Also, the holding medium (the synthetic lake-water) is produced and held in large plastic vats. Thus, it is a complex system of methods and equipment. Although this is a somewhat frustrating fact of our laboratory work, i.e. that our laboratory environment has numerous potential sources of leachates, we believe that many or most laboratory researchers are in the same situation. Recently other researchers have reported on parallel examples of such unfortunate situations, which seem to be difficult to avoid^8^,^9^.

We have found it relevant to highlight initial findings on biological effects from plasticizers and other additives in plastic materials. Although these findings date back as far as 1960’ and 1970’, we have found several of these older papers worthy of revival and reconsideration, as they describe phenomena and pollutants which were seen affecting human health and environmental quality and which are still relevant in our time. Thus we have presented results from several of these older papers throughout this work. Those pioneering researchers partly invented new methods to investigate the issues concerning toxic effects from plastics. As an example, Jaeger & Rubin investigated mobilisation and migration of phthalates from PVC blood transfusion bags into donor blood and recipient tissue^4^. The influx of phthalate based chemicals to stored blood was estimated at 2.5 mg/l/day, resulting in patients of open surgery situations receiving quantities of phthalates large enough to map flux and calculate phtalate mass balances. Also, such mass-balance calculations in one patient indicated amount of phthalates excreted in urine exceeding estimated intake through donor blood, this being attributed to additional leaching from the plastic tubing used in that particular case^4^.

It is thus well documented that chemical constituents in medical plastics such as blood transfusion bags can leach into and contaminate biological material such as mammalian blood. This issue is still important as demonstrated by recent research which describes techniques for extraction and quantification of di(2-ethylhexyl)phthalate (DEPH) as practical methods for safety-assessment of medical PVC plastics, notably bags for transfusion blood^31^. The methods are intended to facilitate identification of suboptimal medical equipment, which is perceived as an important challenge in some hospitals in China.

In the pioneering days of research on effects of plastic materials, scientists also investigated leaching of plastic chemicals from different types of water tubing, such as municipal water-mains and garden hoses made from polypropylene (PP), polyethylene (PE) and polyvinylchloride (PVC) plastics. It was found that water which had flowed through these types of tubing materials contained plasticizer-contamination in concentrations ranging up to 5000 ppb^1^. It should also be noted that the question of whether plastics should be cleaned before use is not new; in 1976 a report on effects of leachates from plastics in aquaculture-systems recommended that such materials should be flushed with warm water for 10 days prior to use^32^.

Although our parent institution medical laboratories is a relatively small facility, the annual consumption of disposable tubes is substantial, averaging 19 000 units of 50 ml-CPPT tubes in each of the three years 2012–2014. The implication is that we not only recommend researchers to consider re-use of their disposable plastics by washing, but also see a potential for saving resources and reducing consumption, when feasible.

Although our focus has been on potentially confounding biological effects, we see that the challenges of these chemical leachates can be even more prominent and have direct human health implications, when the utensils in question are medical devices. A recent study by Serbian researchers presents a good overview of the present challenges with uncontrollable leachate of phthalates from various medical plastics made of polyvinylchloride (PVC) and low-density polyethylene (LDPE)^29^. The researchers found potentially motile DEPH contents of up to 340 mg/g in PVC utensils but considerably lower in LDPE. The authors observed that the softer materials (flexible tubes, bags) leached the highest levels of DEPH. The authors conclude that continuing quality control of such plastic utensils is essential to avoid excessive human exposure. Reviewing this new evidence in the context of the historical evidence of documented negative effects of plastics in the mid-60’s and early 70’s, we find it is astonishing that this problematic phenomenon still persists, half a century after its discovery.

In 1964 the Swedish medical doctor Bengt Gullbring described toxicity problems associated with the use of plastics in blood transfusion equipment, stating that; “*there has been a rapid development in the field of the chemistry of plastics and new plastic materials or ingredients are frequently introduced. Consequently new material is sometimes recommended even if the experience of its use in medicine is limited and few or no toxicological studies have been performed*”^33^. That was written half a century ago and we would have been pleased to announce that those reflected and precautionary words of wisdom by Dr. Bengt Gullbring are no longer relevant. However, unfortunately, they are.

## Conclusion

Significant delay of maturation as well as significant reduction of growth and fecundity was registered in individuals of the model organism *D. magna* which were reared in aqueous solution within the microenvironment of new laboratory-standard 50 ml-CPPT tubes, compared to controls reared in washed and re-used tubes. The results indicate that the “newness” of 50 ml-CPPT tubes represents a toxic factor, which is negatively affecting the life-history of *D. magna.* Plastic “Newness” may thus also cause negative effects in other test species or systems. This needs further investigation.

We observe that the commercial producer of one of the tested brands of laboratory plastics has presented advertising reassurances regarding material quality, which must be revised.

From our experiments, it is not possible to identify specific causal factors responsible for the observed outcomes. However, based on the reviewed literature it is not unreasonable to speculate that chemical leachates consisting of esters of phthalic acid (phthalates), are a possible explanatory factor and thus good candidates for follow-up studies. Nevertheless, also other constituents of polypropylene should be studied because of the relatively low concentration of phthalates in polypropylene and their low solubility.

The results are in accordance with numerous previously published findings on medical, biological and biochemical effects of leachates from plastic materials. Based on those numerous findings in general and the present results in particular it is recommended that laboratory professionals and researchers uphold their awareness of these issues. We should bear in mind that plastic-ware for laboratory research and medical applications has posed toxicological challenges for more than half a century and still demonstrates toxic properties which may influence research results. Toxic effects can be caused by partly unknown biochemical contaminants which might be alleviated by aeration and washing. Publications of experimental work involving plastic utensils should inform on a) type of material (ideally specifying details such as commercial brand) and b) whether the utensils are new or washed/aerated.

Possibly it could cause less disruption to a sensitive experiment to use washed and re-used plastics such as polypropylene tubes, than to trust new tubes fresh out of a box.

## Additional Information

**How to cite this article:** Cuhra, M. *et al*. *In plastico*: laboratory material newness affects growth and reproduction of *Daphnia magna* reared in 50-ml polypropylene tubes. *Sci. Rep.* **7**, 46442; doi: 10.1038/srep46442 (2017).

**Publisher's note:** Springer Nature remains neutral with regard to jurisdictional claims in published maps and institutional affiliations.

## Figures and Tables

**Table 1:** Selected references from past 5 decades (1964–2016) that investigate chemical leachates from plastic materials used in laboratory, medical and household purposes.

Study	Subject/material	Findings	Biological effect
Gullbring^33^; Gullbring^3^ *et al*.	Medical: plastic for blood transfusion	Leachates; Toxicity test of water-soluble leachates in cell cultures	Cytotoxicity, potential for contamination of blood for transfusion purposes
Jaeger & Rubin^4^	Medical: PVC-blood bags	Leachate of phthalates from PVC plastic	Phthalate contamination found in patients
Mayer & Sanders^2^	Biology: Toxicological study of phthalates in aquatic organisms	Modelling leachate accumulation in aquatic organisms,	Reproductive toxicity in *D. magna*
Junk *et al*.^1^	Chemistry: PE, PP, PVC pipes for water transportation.	Leachates; chromatographic/spectroscopic detection of various organic contaminants/polymer additives/plasticizers	Not reported
Guess & Haberman^34^	Biochemistry; PVC and polyolefinic plastics	Leachates from PVC	Eosinophilic/toxic response
Mcdonald *et al*.^5^	Medical biology; Disposable tubes and microwell-plates	Leachates of bioactive chemicals identified from investigated plastics	Inhibition of human-analogue enzyme (monoamine oxidase-B)
Belaiche *et al*.^35^	Medical biology; PP-pipette tips	Leachates identified as nonylphenol ethoxylate	Inhibition of NADH-coenzyme Q reductase
Watson *et al*.^36^	Medical biology; Disposable 50 ul pipette tips	Leachates identified as erucamide	Not reported, biological activity assumed
Lewis *et al*.^37^	Biochemistry; PP-microtubes	Leachates identified as complex mixture of leached macromolecules	No specific effects reported but authors speculate that ”wide-spread problems exist”
Xu *et al*.^14^	Biochemistry; 50 ml PP-tubes	Leaching chemicals causing changes in spectroscopy of contained liquids	Not reported
Ito *et al*.^38^	Biochemistry; 21 different plastics, PP, PE, PVC butylrubber etc.	Leachates causing inhibition of calcineurin phosphatase	Not reported
Swanson *et al*.^7^	Medical; Disposable syringes	Adhesion in plastic material of active pharmacological ingredient	Reduced delivery of specific agent in patients
Marx^6^	Biochemistry; Review, interview w. producers	Leaching of chemical slip-agents added as surfactants in labware and added to PP to make it transparent	Laboratory experiments adversely affected
Kostic^29^	Medical PVC and LDPE	High levels of DEPH phthalates leak from new plastics.	Not reported
